# Anticonvulsant Effects of Fractions Isolated from *Dinoponera quadriceps* (Kempt) Ant Venom (Formicidae: Ponerinae)

**DOI:** 10.3390/toxins9010005

**Published:** 2016-12-23

**Authors:** Diana Aline Morais Ferreira Nôga, Luiz Eduardo Mateus Brandão, Fernanda Carvalho Cagni, Delano Silva, Dina Lilia Oliveira de Azevedo, Arrilton Araújo, Wagner Ferreira dos Santos, Antonio Miranda, Regina Helena da Silva, Alessandra Mussi Ribeiro

**Affiliations:** 1 Physiology Department, University Federal of Rio Grande of Norte, Natal, RN 59078-970, Brazil; dy_2012@hotmail.com (D.A.M.F.N.); lemb.89@gmail.com (L.E.M.B.); nandacagni@hotmail.com (F.C.C.); delanoanibal@gmail.com (D.S.); dinalillia@gmail.com (D.L.O.d.A.); arrilton@gmail.com (A.A.); 2 Biology Department, University of São Paulo, Ribeirao Preto, SP 14040-901, Brazil; wagnerf@usp.br; 3 Biophysics Department, Federal University of São Paulo, São Paulo, SP 04023-062, Brazil; miranda.unifesp@gmail.com; 4 Pharmacology Department, Federal University of São Paulo, São Paulo, SP 04023-062, Brazil; reginahsilva@gmail.com; 5 Biosciences Department, Federal University of São Paulo, Santos, SP 11015-020, Brazil

**Keywords:** ant venom, neuroactive compounds, bicuculline, tonic-clonic seizures, peptide fraction, natural product

## Abstract

Natural products, sources of new pharmacological substances, have large chemical diversity and architectural complexity. In this context, some toxins obtained from invertebrate venoms have anticonvulsant effects. Epilepsy is a neurological disorder that affects about 65 million people worldwide, and approximately 30% of cases are resistant to pharmacological treatment. Previous studies from our group show that the denatured venom of the ant *Dinoponera quadriceps* (Kempt) protects mice against bicuculline (BIC)-induced seizures and death. The aim of this study was to investigate the anticonvulsant activity of compounds isolated from *D. quadriceps* venom against seizures induced by BIC in mice. Crude venom was fractionated by high-performance liquid chromatography (HPLC) resulting in six fractions referred to as DqTx1–DqTx6. A liquid chromatography-mass spectrometry (LC/MS) analysis revealed a major 431 Da compound in fractions DqTx1 and DqTx2. Fractions DqTx3 and DqTx4 showed a compound of 2451 Da and DqTx5 revealed a 2436 Da compound. Furthermore, the DqTx6 fraction exhibited a major component with a molecular weight of 13,196 Da. Each fraction (1 mg/mL) was microinjected into the lateral ventricle of mice, and the animals were observed in an open field. We did not observe behavioral alterations when the fractions were given alone. Conversely, when the fractions were microinjected 20 min prior to the administration of BIC (21.6 nM), DqTx1, DqTx4, and DqTx6 fractions increased the latency for onset of tonic-clonic seizures. Moreover, all fractions, except DqTx5, increased latency to death. The more relevant result was obtained with the DqTx6 fraction, which protected 62.5% of the animals against tonic-clonic seizures. Furthermore, this fraction protected 100% of the animals from seizure episodes followed by death. Taken together, these findings indicate that compounds from ant venom might be a potential source of new anticonvulsants molecules.

## 1. Introduction

Natural products have a wide chemical diversity and architectural complexity that cannot be matched by synthetic molecules [1,2]. Animal venoms stand out because of the high specificity and potency of their toxins in molecular targets of mammalian biological systems [1]. These venoms can exert noxious effects on several organic systems, such as cardiovascular, nervous, respiratory, and renal systems, as well as skin and muscles. As a consequence, poisoning victims can experience pain, swelling, tissue necrosis, vomiting, paralysis, fever, diarrhea, headaches, blurred sight, dizziness, hypotension, hemorrhage, and even death [3,4]. From another standpoint, some venoms have beneficial effects [5,6] or can be used as pharmacological tools for probing biochemical pathways and mechanisms [7,8]. Despite the remarkable potential of venoms, their evaluation and characterization remains underexplored.

Invertebrates have incorporated a vast range of neurotoxins in their venoms throughout evolution, and some compounds show high affinity to receptors, ionic channels, and transporters in the central nervous system (CNS) [7,8,9,10]. Previous studies have demonstrated the anticonvulsant effects of toxins isolated from invertebrate venoms. The peptide fraction (350 μg/animal) isolated from the venom of the wasp *Polybia paulista* protected 60% of the rats against generalized tonic-clonic seizures induced by pentylenotetrazol (PTZ) [11]. Furthermore, parawixin 2 (isolated from the venom of the spider *Parawixia bistriata*) protected animals against seizures induced by PTZ, picrotoxin, pilocarpine, and kainic acid [12], as well as inhibited PTZ-induced kindling of rats [13].

Seizures are the main symptoms of epilepsy, a neurological disorder characterized by an enduring predisposition to transient abnormal excessive or synchronous neuronal activity. Patients also present neurobiological, cognitive, psychological, and social consequences of the condition [14,15]. This disorder affects about 65 million people worldwide [16] and approximately 30% of patients are resistant to pharmacotherapy [17,18]. Furthermore, antiepileptic drugs frequently cause relevant side effects that range from gastric discomfort to hepatic failure or aplastic anemia [9]. In this context, invertebrate venoms are a possible source for new anticonvulsant probes [19].

Recently, studies have shown anticonvulsant effects of the crude venom from the giant ant *Dinoponera quadriceps*. Lopes et al. [20] demonstrated that intraperitoneal administration of the crude venom increased the latency for onset of PTZ-induced seizures in mice. Additionally, our group observed procursive behavior and tonic-clonic seizures after injecting the crude venom into the lateral ventricle of mice. In contrast, prior administration of the denatured venom protected the animals against tonic-clonic seizures (66.7%) and death (100%) induced by the gamma aminobutyric acid GABAA antagonist bicuculline (BIC) [21]. Taken together, these findings demonstrate that *D. quadriceps* venom is a potential source of new pro- and anticonvulsants molecules. The aim of the present study was to investigate the anticonvulsant activity of isolated fractions of *D. quadriceps* venom on seizures induced by BIC in mice. We will also verify the effects of the fractions on spontaneous behavior.

## 2. Results

### 2.1. HPLC Fractionation and Mass Spectra

**Figure 1:**
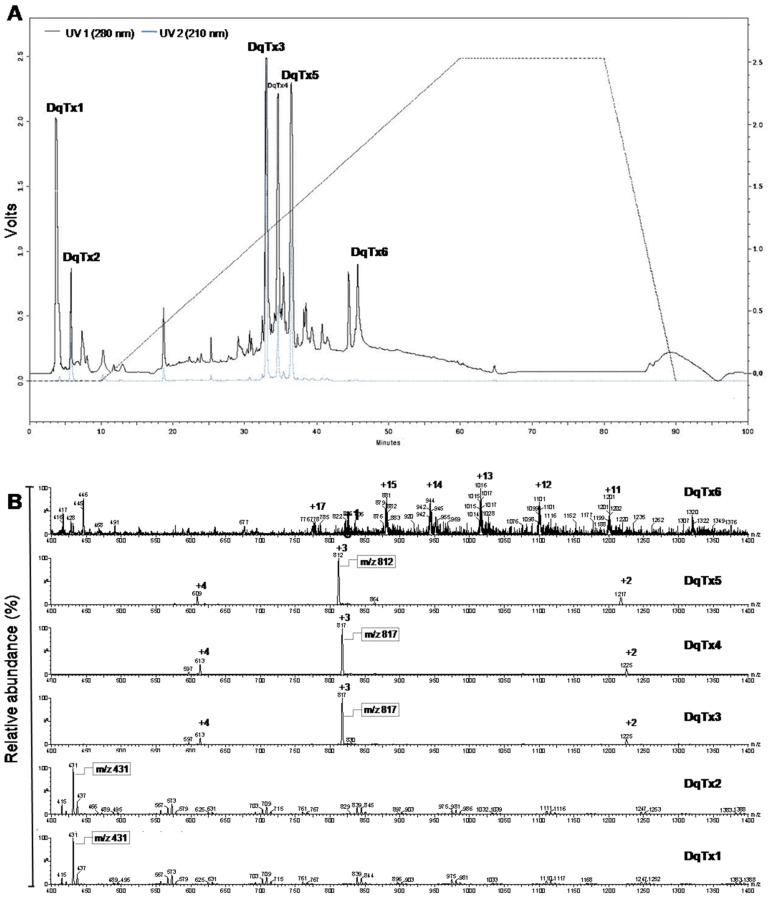
*Dinoponera quadriceps* crude venom chromatography. (**A**) Chromatographic profile (reverse-phase high performance liquid chromatography—Hitachi system, Phenomenex C18 column 2.6 × 25 cm, 12 µm, 300 Å) of crude *Dinoponera quadriceps* venom, showing six major fractions monitored at 210 (gray) and 280 (black) nm and eluted using a linear gradient from acetonitrile containing trifluoroacetic acid at 0.1% (TFA) (100% ACN/H_2_O *v*/*v*) for 100 min; (**B**) ESI mass spectrum (LC/ESI-MS—Waters system, mass range between 200 and 2000 *m*/*z*, nitrogen gas flow rate of 4.1 L·h^−1^, capillary voltage of 2.3 kV, cone voltage of 32 V, extractor voltage of 8 V, source heater set at 100 °C, solvent heater set at 400 °C, ion voltage of 1.0 V, and a multiplier voltage of 800 V) of the fractions of DqTx1 to DqTx6.

**Figure 2:**
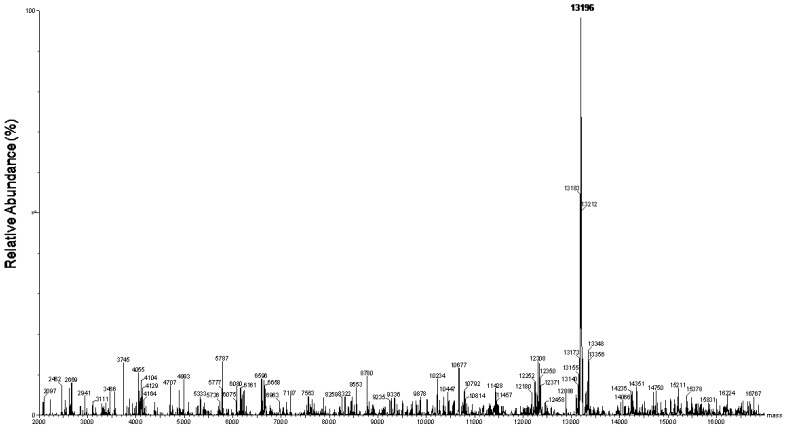
Deconvolution electrospray mass spectrum of fraction DqTx6.

The chromatographic profile of the crude venom is shown in Figure 1A. This profile revealed the presence of the six fractions that were denoted as DqTx1 to DqTx6. The fractions were lyophilized and used in the biological assays. Each fraction was analyzed by liquid chromatography/eletrospray ionization-mass spectrometry (LC/ESI-MS). The fractions were monitored at 210 and 280 nm. The chromatogram revealed initial elution time of 3 and 7 min to fractions DqTx1 and DqTx2, respectively. ESI-MS spectra of both fractions displayed a major compound with molecular weight of 431 Da. DqTx3 and DqTx4 fractions had elution times of 34 to 35 min with a major compound of 2421 Da. Retention time of fraction DqTx5 ranged from 36–38 min with a major compound of 2436 Da (Figure 1). Furthermore, DqTx6 presented an elution time range from 46–48 min, and the deconvolution mass spectrum revealed a major compound with molecular weight of 13,196 Da (Figure 2).

### 2.2. Primary Behavioral Screening

One-way ANOVA did not reveal effect of i.c.v. treatment with fractions of DqTx1 to DqTx6 for exploratory activity (*p* = 0.49), grooming (*p* = 0.07), or immobility (*p* = 0.679) (Table 1). These fractions did not induce motor or behavioral alterations in the animals.

### 2.3. Anticonvulsant Assay

As expected, all animals that received vehicle prior to bicuculline administration showed tonic-clonic seizures (level 5) followed by death (Table 2).

One-way ANOVA revealed effects in latency to onset of seizures (*p* = 0.029) and latency to death (*p* = 0.004) (Table 2). Dunnett’s post hoc analysis detected that groups pretreated with DqTx1 (*p* = 0.008), DqTx4 (*p* = 0.044), and DqTx6 (*p* = 0.002) showed an increase in the mean latency for the onset of seizures, and for the effect of DqTx3 was marginally significant (*p* = 0.057) (Table 2). While the control group presented mean latency of 16.5 ± 6.8 s, DqTx1, DqTx3, DqTx4, and DqTx6 had mean latencies of 1191.14 ± 306.27, 879.28 ± 310.05, 922.86 ± 318.62 and 1225.28 ± 273.44 s, respectively. Similar results occurred regarding the latency for death; groups that received pretreatment with DqTx1 (*p* = 0.005), DqTx2 (*p* = 0.009), DqTx3 (*p* = 0.01), DqTx4 (*p* = 0.025), and DqTx6 (*p* < 0.001) showed increased latency for death (Table 2). While the control group had a mean latency of 509.25 ± 253.24, DqTx1, DqTx2, DqTx3, DqTx4, and DqTx6 had mean latencies of 1528.71 ± 180.29, 1471.86 ± 169.40, 1453.71 ± 198.99, 1342.71 ± 257.40, and 1800, respectively.

The analysis of seizures score showed that pretreatment with DqTx1, DqTx4, and DqTx5 fractions prevented the development of tonic-clonic seizures (level 5) in 42.6% of animals. DqTx3 and DqTx6 fractions protected 28.6% and 62.5% of animals, respectively. Regarding survival, the pretreatment with DqTx2 prevented the death of 42.8% of animals, DqTx4 and DqTx5 prevented the death of 57.1% of animals. DqTx1 and DqTx3 protected 71.4% and DqTx6 protected 100% of the animals (Table 2).

## 3. Discussion

RP-HPLC was a suitable technique to fractionate the *Dinoponera quadriceps* crude venom (Figure 1A). The separated fractions are most likely composed of peptides and a small protein. The ESI-MS spectra revealed that the DqTx1/DqTx2 fractions contained hydrophilic compounds of low molecular weight. The DqTx 3 and DqTx4 fractions presented the same composition as the hydrophobic 2451 Da molecular weight compound. Interestingly, the DqTx5 fractions revealed a hydrophobic compound with a slightly higher molecular weight of 2436 Da. Furthermore, the DqTx6 fraction contained a major hydrophobic compound with a molecular weight of 13,196 Da. This fraction showed more potent anticonvulsant activity when compared with the other fractions (Table 2), and will be fractionated further in the future.

Peptides are the most common compounds found in animal venoms, and represent a main source of bioactive toxins with high affinity and selectivity for a range of targets [9,22]. Venoms from the ant subfamilies Ponerinae and Myrmeciinae are particularly rich in peptides with antimicrobial properties, such as ponericins [23], pilosulins [24], and some dinoponeratoxins [25]. Moreover, there are other peptides from ant venoms of these subfamilies, such as poneratoxins and poneritoxins, respectively. Poneratoxins are V-shaped peptides with two α-helices connected by a β-turn, which modulate voltage-gated sodium channels and block synaptic transmission in the insect CNS [22,26,27,28,29]. Poneritoxins are monomeric peptides with one or two disulfide bonds, which inhibit human L-type voltage-gated calcium channels and have insecticidal activity [30].

Several peptides that were isolated from invertebrate venoms have anticonvulsant properties. BmK IT2 and BmK AS are β-type toxins isolated from the scorpion *Buthus martensis* venom, which increase the latency for onset of seizures and reduce mortality induced by the administration of PTZ when injected into the rat hippocampus [31,32]. Similarly, CGX-1007 isolated from the cone snail *Conus geographus* venom blocks maximal electroshock, audiogenic, threshold tonic extension, PTZ, picrotoxin, and BIC-induced seizures when injected into the lateral ventricle of mice [33], and reduces the amygdala-kindled seizure score in rats [34]. Moreover, the ω-agatoxin IVA is a voltage-sensitive calcium channel blocker isolated from the spider *Agelenopsis aperta* venom that blocks audiogenic seizures when injected into the lateral ventricle of mice [35]. A peptide fraction isolated from the *Polybia paulista* wasp venom increases latency for the onset of seizures and protects 60% of the animals from tonic-clonic seizures induced by PTZ in rats [11].

Bicuculline (BIC) is a competitive GABA_A_ receptor antagonist that is well established as a model for the study of seizures in rodents and scanning potential anticonvulsant drugs [36,37,38,39]. Despite of the extreme effectiveness of the BIC (all animals show severe tonic-clonic seizures followed by death), it is suitable for screening of potentially anticonvulsant substances from natural products [37,38,39,40,41].

In the present study, all fractions protected animals from BIC-induced seizures, death, or both. For example, DqTx1, DqTx3, and DqTx6 fractions protected animals from seizures (42.8%, 28.6%, and 62.5%) and death (71.4%, 71.4%, and 100%), respectively. Notwithstanding, it should be pointed out that all the fractions demonstrated some degree of anticonvulsive effects. In this respect, an unidentified factor related to the generation of HPLC fractions could be responsible for the anticonvulsive effects. However, the fractionation technique applied here has been extensively used, and no study reported an association between the fractionation process and the anticonvulsive action [39,42,43,44] or other pharmacological effects [45,46,47,48,49,50,51]. Furthermore, the fact that all of the fractions have anticonvulsant potential is surprising, but not unlikely. Indeed, venoms can have more than one effective compound regarding a certain pharmacological action [31,32,52,53,54,55,56,57,58]. Additionally, as reported in the results session, the effects were not homogeneous among the fractions, as it would be expected if the fractionation processes were responsible for the pharmacological activity.

Antiepileptic drugs act by reducing the neuronal excitability through three main mechanisms: modulating voltage-dependent ion channels, decreasing the excitatory transmission, or increasing inhibitory neurotransmission mediated by GABA or glycine [59]. Voltage-dependent ion channels (Na^+^, Ca^2+^ or K^+^) play a crucial role in the action potential and consequently in neurotransmission. Hence, some venom toxins with anticonvulsant potential target voltage-dependent channels. For example, the ω-conotoxins MVIIA and GVIA are N-type calcium channels antagonists [60], whereas the α-type neurotoxins BmK AS and BmK IT2 are sodium channel modulators [31,32].

l-glutamate receptors and transporters are logical targets for anticonvulsant drugs, as they mediate the majority of excitatory synapses in the human brain. Parawixin 10, a toxin extracted from *P. bistriata*, exerts its anticonvulsant activity by increasing glutamate uptake [44]. However, this mechanism is not common for antiepileptic drugs due to the wide range of side effects [9,59,61].

Several antiepileptic drugs act on the GABA_A_ receptor as direct agonists or increase GABA availability in the synaptic cleft by blocking uptake [62,63,64]. The toxins SrTx1 and FrPbA2, isolated from the venom of the spiders *Scaptocosa raptoria* and *P. bistritata*, respectively, act via GABAergic mechanisms [65].

In the last few years, some studies have demonstrated the pharmacological properties of *D. quadriceps* venom. Sousa et al. [66] injected different concentrations of crude venom endovenously (e.v). 30 min before nociceptive tests (formalin, writhing and hot plate tests). The venom showed antinociceptive effects by inhibiting the second phase of the nociceptive response caused by formalin injection, reducing writhing induced by acetic acid and increasing latency of the response to thermal stimuli. Cologna et al. [25] evaluated the antimicrobial activity of four peptides from the venom, and two of them showed activity against Gram-positive and -negative bacteria. Corroborating those data, Lima et al. [67] reported the antimicrobial activity of the crude venom against methicillin-sensitive and resistant *Staphylococcus aureus* strains. The same researchers also reported the antiparasitic effects of the venom against *Leishmania amazonensis* and *Trypanosoma cruzi* [68].

Recently, other studies [69,70] tested the inflammatory activity of the venom using the paw edema and peritonitis models, showing a dose-dependent inflammatory response in the paw edema model and an increase in the number of leucocytes and interleukin-1β in the peritoneal fluid. In addition, the venom also showed anticoagulant and antiplatelet effects in vivo [69].

Regarding the anticonvulsant property, Lopes et al. [20] tested the crude venom through intraperitoneal (i.p.) and endovenous e.v. routes against three different chemoconvulsants (PTZ, pilocarpine, and strychnine). As a result, mice that received the venom e.v. before PTZ administration presented a reduced latency to the first seizure. Conversely, mice that received the venom i.p. previously to PTZ had an increase in this latency. No effects of the venom were observed in animals that received pilocarpine or strychnine. These results indicated that *D. quadriceps* venom has compounds with convulsant and anticonvulsant properties, which was corroborated in a previous study from our group [21]. The fractions used herein showed a higher increase in latency to the onset of seizures, and also promoted increases in the latency to death, in survival, and protection against tonic-clonic seizures compared to those of Lopes et al. [20]. This difference is probably due to the administration route. We injected directly into the brain, whereas the venom was systemically administered (e.v. or i.p.) in the other study. Furthermore, as reported by Cologna et al. [25], there were different peptide compositions of *D. quadriceps* venom samples collected from four different regions in Bahia state, with more similarity observed between samples from the closest areas. Thus, our results may be different from these of Lopes et al. due to changes in the composition of venom influenced by the environment.

In summary, in the present work the DqTx fractions protected animals from seizures and death elicited by a GABA_A_ antagonist, and Lopes et al. [20] showed protection against another GABA_A_ antagonist, but not against drugs acting through other pathways. Thus, we suggest that the anticonvulsant activity of the DqTx fractions involves GABAergic transmission. Indeed, recent transcriptome [71] and peptidomic [25] analyses of the *D. quadriceps* venom revealed the presence of peptides with similar structures to toxins that interact with ion channels. In addition, mass spectrometry results showed that the fractions contained low molecular weight substances, i.e., < 2550 Da (DqTx1-DqTx5) and 13,169 Da (DqTx6). However, the mechanism underlying the anticonvulsant effects of the DqTx fractions requires further investigation.

In conclusion, *D. quadriceps* venom is an important source of novel compounds. Our results indicate that the low molecular weight fractions from ant venom are promising anticonvulsants for experimental pharmacology. Taken together, these findings will help determine the biological effects of ant venom components on the mammalian CNS. Further studies are needed to determine the molecular structures and improve understanding of their effects and therapeutic potential.

## 4. Material and Methods

### 4.1. Ant Collection

*D. quadriceps* specimens were collected in Nísia Floresta (6°5′ S, 35°12′ W), Rio Grande do Norte state, Brazil. Specimens were frozen at −20 °C and venom reservoirs were dissected.

### 4.2. Fractionation and Mass Spectrometry Analysis

Crude content of two hundred venom reservoirs were lyophilized and diluted in 1 mL of trifluoroacetic acid at 0.1% (TFA/H_2_O). This solution was submitted to high performance liquid chromatography (HPLC; Hitachi, Tokyo, Japan) fractionation using a Phenomenex C18 reverse phase column (2.6 × 25 cm, 12 µm, 300 Å; Sigma, St. Louis, MO, USA). Elution was carried out with 0.1% TFA/H_2_O at a 100% gradient for the first 10 min, followed by a linear gradient from 0% to 100% acetonitrile (ACN) containing 0.1% TFA for 100 min. Eluates were monitored at 210 and 280 nm and the main fractions collected were lyophilized and resuspended in 1 mL of distilled water. Six major fractions were obtained and named DqTx1–DqTx6.

HPLC fractions were analyzed by liquid chromatography electrospray ionization mass spectrometry (LC/ESI-MS). LC/ESI-MS data were obtained from a Waters instrument (model 3100, Milford, MA, USA) coupled to a Waters Alliance system (model 2695) using a C18 column (2.0 × 150 mm, 3.0 µm particle size, 110 Å pore size, Phenomenex Gemini, Torrance, CA, USA). Solvent A was 0.1% TFA in water, and solvent B was 60% ACN in solvent A. The gradient was 5%–95% in solvent B for 30 min, and the peptides were detected at 220 nm. Mass measurements were performed in a negative mode with the following conditions: mass range between 200–2000 *m*/*z*, nitrogen gas flow rate of 4.1 L·h^−1^, capillary voltage of 2.3 kV, cone voltage of 32 V, extractor voltage of 8 V, source heater set at 100 °C, solvent heater set at 400 °C, ion voltage of 1.0 V, and a multiplier voltage of 800 V.

### 4.3. Animals

Three-month-old male Swiss mice (30–50 g) were housed with free access to food and water, with 5–6 animals in plastic cages (20 × 30 × 13 cm), under conditions of controlled temperature (25 ± 1 °C) and a 12 h light/12 h dark cycle (lights on 6:30 a.m.). Animals were handled in accordance with Brazilian law for the use of animals in research (Law Number 11.794), and all procedures were approved by the local ethics committee (Federal University of Rio Grande do Norte; protocol nr. 035/2010, approved 15 September 2010). All efforts were made to minimize animal potential pain, suffering, or discomfort.

### 4.4. Surgery

Prior to surgery, mice were anesthetized with intraperitoneal ketamine (100 mg/kg) plus xylazine (10 mg/kg). Afterwards, the animals were positioned in a stereotaxic frame (Insight, Ribeirão Preto, Brazil) and skulls were exposed. SA stainless steel guide cannula (25 gauge, 8 mm length) was implanted in the lateral ventricle, and the stereotaxic coordinates were anterior-posterior = −0.6 mm, medial-lateral = 1.1 mm, and dorsal-ventral = 1.0 mm from the bregma [72]. The guide cannula was anchored to the skull with dental acrylic. At the end of the surgery, the cannula was temporarily sealed with a stainless-steel wire to avoid obstruction. Animals were given one week of post-operative recovery prior to the start of the experimental proceedings.

### 4.5. General Procedures

Drug (BIC), vehicle, and chromatography fractions (1 mg/mL) were injected intracerebroventricular (i.c.v.) at a rate of 0.5 µL/min to a final volume of 1 µL via a microsyringe pump (Insight, Brazil) with a 10 µL syringe (Hamilton Co., Reno, NV, USA) connected to an injection needle. After infusion, the needle was left in the guide cannula for an additional 60 s to allow diffusion from the needle tip. Afterwards, mice were placed in a circular open field (30 cm in diameter with wall height of 60 cm) located in an experimental room illuminated by a 40 W fluorescent lamp for 30 min. The behavioral session was recorded by a digital camera placed above the apparatus and behavioral parameters were registered. The apparatus was cleaned with a 5% alcohol solution after each session.

### 4.6. Behavioral Analysis

*General screening:* Animals were randomly assigned to one of the following groups: a control group that received 1 µL of distilled water (CTR, *n* = 9); and six experimental groups that received 1 mg/mL of each fraction (DqTx1, *n* = 7; DqTx2, *n* = 7; DqTx3, *n* = 8; DqTx4, *n* = 8; DqTx5, *n* = 8; DqTx6, *n* = 8). We quantified the time spent in the following behavioral clusters: exploration (exploration activities such as exploratory sniffing, walking, scanning, and erect posture); grooming (grooming of head, snout, claws, and back); and immobility (complete absence of movements, except for respiratory movements).

*Anticonvulsant Assay:* The GABA_A_ receptor antagonist bicuculline methbromide (BIC, 10 mg/mL i.e., 21.6 nM, Sigma, USA) was standardized to provoke tonic-clonic seizures in 100% of the animals in less than 30 min. Animals were randomly assigned to one of the following groups: a control group (CTR, *n* = 8) that was microinjected with vehicle 20 min prior to bicuculline administration; and six experimental groups that received the fractions DqTx1 to DqTx6 twenty min prior to bicuculline administration (DqTx1, *n* = 7; DqTx2, *n* = 7; DqTx3, *n* = 7; DqTx4, *n* = 7; DqTx5, *n* = 7; DqTx 6, *n* = 8). Immediately after the administration of BIC animals were placed in the open field and behavior was registered for 30 min.

The severity of seizures was evaluated using an adapted Racine’s scale [73], as follows: (1) myoclonic jerks of contralateral paw; (2) mild paw clonus lasting at least 5 s; (3) severe paw clonus lasting at least 15 s; (4) rearing in addition to severe paw clonus; and (5) rearing and falling in addition to severe paw clonus. Moreover, latency to the onset of tonic-clonic seizures (score 5) and death, survival, and percentage of protection against tonic-clonic seizures were evaluated.

### 4.7. Verification of the Injection Site

Upon completion of the behavioral procedures, mice were euthanized with intraperitoneal injection of sodium thiopental (70 mg/kg) and microinjected into the left lateral ventricle with 1.0 µL of methylene blue stain to mark the site of injection. Brains were removed and manually cut to check the position of the cannula. Only animals with correct injection sites were included in the analysis. The same procedure was held if the death occurred before the end of the experiments.

### 4.8. Statistical Analysis

Data normality and homogeneity of variances were tested by Shapiro-Wilk and Levene tests, respectively. Comparisons of the behavioral clusters among groups treated with the different fractions of the venom were held by one-way analysis of variance (ANOVA) followed by Dunnett’s post hoc (one-tailed). The same tests were used to compare latencies for the onset of seizure and death. The number of protected animals in the anticonvulsant assays was analyzed using *χ*^2^ test, followed by residual analysis. Differences between medians of score in these assays were analyzed using the Mann-Whitney test with Bonferroni correction. We considered *p* < 0.05 as significant values. All statistical analyses were conducted with PASW Statistics 22 software (IBM, Armonk, NY, USA).

## Figures and Tables

**Table 1:** Effects of injection into the lateral ventricle of fractions of *Dinoponera quadriceps* venom on the total time spent in exploratory activities, grooming, and immobility in mice.

Treatment	Behavioral Cluster (s)		
	*Exploration*	*Grooming*	*Immobility*
Control (*n* = 9)	1395.48 ± 56.38	293.14 ± 55.36	111.36 ± 36.47
DqTx1 (*n* = 7)	1281.37 ± 110.25	352.47 ± 104.24	166.15 ± 93.05
DqTx2 (*n* = 7)	1250.36 ± 128.08	267.05 ± 58.79	282.58 ± 119.69
DqTx3 (*n* = 8)	1367.91 ± 58.9	294.31 ± 37.89	137.77 ± 53.78
DqTx4 (*n* = 8)	1193.58 ± 49.32	493.80 ± 50.81	112.61 ± 44.11
DqTx5 (*n* = 8)	1250.40 ± 87.55	406.00 ± 38.47	143.59 ± 62.27
DqTx6 (*n* = 8)	1378.41 ± 84.41	280.44 ± 43.68	141.14 ± 64.03

Data expressed as the mean ± SEM. *p* > 0.05 (One-way ANOVA).

**Table 2:** Effects of injection into the lateral ventricle of *Dinoponera quadriceps* venom fractions against seizures elicited by the bicuculline model in mice.

Parameters	Treatment						
	Control	DqTx1	DqTx2	DqTx3	DqTx4	DqTx5	DqTx6
Median seizure score	5	5	5	5	5	5	4
Incidence of seizures							
Stage 1	0/8	6/7	4/7	3/7	5/7	3/7	5/8
Stage 2	0/8	4/7	4/7	3/7	5/7	3/7	5/8
Stage 3	0/8	2/7	5/7	2/7	3/7	2/7	4/8
Stage 4	0/8	1/7	2/7	2/7	0/7	1/7	1/8
Stage 5	8/8	4/7	7/7	5/7	4/7	4/7	3/8
Percentage of protection	0	42.8	0	28.6	42.8	42.8	62.5 ^#^
Percentage of survival	0	71.4	42.8	71.4	57.1	57.1	100 ^#^
Latency for the onset of seizures (s)	16 ± 6	1191 ± 306 *	710 ± 216	879 ± 310 ^+^	923 ± 318 *	812 ± 350	1225 ±273 *
Latency for death (s)	509 ± 253	1528 ± 180 *	1471 ± 169 *	1453 ± 199 *	1342 ± 257 *	1087 ± 337	1800 *
Incidence of death	8/8	2/7	4/7	2/7	3/7	3/7	0/8

Bicuculline was injected into the lateral ventricle at a dose of 10 mg/mL following pre-treatment with vehicle or fractions. ^#^ *p* < 0.05 compared to control (chi-square, followed by residual analysis); * *p* < 0.05; and ^+^ *p* = 0.057 compared to control (one-way ANOVA followed by Dunnett’s post hoc test).
